# Cascading Adjacent Level Vertebral Compression Fractures Necessitating a Series of Eleven Kyphoplasties

**DOI:** 10.1155/2015/395875

**Published:** 2015-10-05

**Authors:** Evan Curatolo, Matthew Reuter, Adil Samad, Daniel Flynn, Marc Menkowitz, Steve Paragioudakis

**Affiliations:** ^1^Department of Orthopeadic Surgery, Monmouth Medical Center, 300 Second Avenue, Room 251SW, Long Branch, NJ 07740, USA; ^2^Jersey Shore University Medical Center, 1945 Route 33, Neptune, NJ 07753, USA

## Abstract

Vertebral kyphoplasty is a procedure used for the treatment of compression fractures. While early randomized-controlled trials were equivocal regarding its benefits, more recent RCTs have shown favorable results for kyphoplasty with regard to pain relief, functional recovery, and health-care related quality of life compared to control patients. Risks of kyphoplasty include but are not limited to cement extrusion, infection, hematoma, and vertebral body fracture of adjacent levels. We describe a case of a 66-year-old male attorney who underwent eleven kyphoplasties in an approximately one-year period, the majority of which were for fractures of vertebrae adjacent to those previously treated with kyphoplasty. Information on treatment was gathered from the patient's hospital chart and outpatient office notes. Following the last of the eleven kyphoplasties (two at T8, one each at all vertebrae from T9 to L5), the patient was able to function without pain and return to work. His physiologic thoracic kyphosis of 40 degrees prior to the first procedure was maintained, as were his lung and abdominal volumes. We conclude that kyphoplasty is an appropriate procedure for the treatment of vertebral compression fractures and can be used repeatedly to address fractures of levels adjacent to a previous kyphoplasty.

## 1. Introduction

Vertebral kyphoplasty is a procedure used for the treatment of compression fractures. It is hoped to alleviate back pain and both restore and maintain vertebral body height. To perform the procedure, a balloon is inserted into the compressed vertebral body and then inflated to reduce the compression fracture. Cement is then introduced into the vertebral body to maintain the height and stabilize the fracture. The clinical benefit of this procedure is currently disputed, with studies demonstrating mixed results.

In 2010, the AAOS established guidelines on the treatment of osteoporotic spinal compression fractures concerning kyphoplasty. These guidelines stated that “kyphoplasty is an option for patients who present with an osteoporotic spinal compression fracture on imaging with correlating clinical signs and symptoms and who are neurologically intact [1].” The strength of this recommendation was limited as the first randomized-controlled trial involving kyphoplasty was not published until 2009. This trial, known as the Fracture Reduction Evaluation (FREE) trial, showed a small but statistically significant increase in SF-36 physical component scores for kyphoplasty patients when compared with nonsurgically managed patients at 1-month follow-up [2]. This recommendation was dampened further by a concurrent recommendation against vertebroplasty, a related procedure in which a less viscous, more liquid cement than that used in kyphoplasty is injected into the vertebral body to restore height and stabilize the fracture without prior insertion of a balloon for fracture reduction. This negative recommendation is based on two randomized-controlled trials published in 2009 that showed little benefit to vertebroplasty over a sham procedure [3, 4].

More recent literature has been more favorable to kyphoplasty. In their 2014 review article [5], Savage et al. note that five of the six randomized-controlled trials of kyphoplasty published since 2009 have shown a benefit of treatment when compared with nonsurgical management. The sixth study showed neither a treatment benefit nor an increased risk to the patient. A long term follow-up of the FREE trial cohort found sustained clinical benefits, most notably improvement of vertebral kyphosis, at 2-year follow-up [6]. Additionally, a 2013 meta-analysis by Anderson et al. showed greater pain relief, functional recovery, and health-care related quality of life in patients undergoing vertebroplasty compared with nonoperatively managed controls [7].

This case report details a 66-year-old male that suffered multiple vertebral compression fractures due to long term steroid use for chronic obstructive pulmonary disease (COPD). He was treated with a total of eleven kyphoplasties, two in T8 and nine in T9-L5, which allowed him to maintain his physiologic kyphosis. Before sustaining any vertebral compression fractures, his thoracic kyphosis was 40°; after completion of all eleven kyphoplasties, his thoracic kyphosis was 38°. This in turn preserved his lung volume and abdominal volume, prevented worsening of his COPD, and allowed him to function without pain and maintain his job. While we know other cases of cascading compression fractures, we are unaware of a similar case documenting eleven consecutive kyphoplasties being described before.

## 2. Case Report

This patient is a 66-year-old male attorney with COPD necessitating the use of 20 mg of prednisone daily for twelve years. A COPD exacerbation resulting in coughing episodes led to the development of persistent and severe back pain. On June 10, 2010, the patient was seen for his initial orthopaedic evaluation. Physical exam was notable for significant tenderness in the midthoracic spine and an obvious kyphotic deformity. MRI documented acute wedge shaped compression fractures at T7, T8, and T9, along with old compression fractures of T5 and L5. There was no cord compression, edema, or retropulsion of elements into the canal. DEXA scan revealed L1–4 *T*-score of −3.1 and left femoral neck *T*-score of −2.4. The patient was referred to Interventional Radiology (IR) for kyphoplasty, fitted for a brace, and scheduled for follow-up in six weeks.

One month later, the patient underwent kyphoplasty of T8 and T9. He experienced significant relief of back pain following the procedure. However, two weeks later, he began to experience back pain again. He was seen by the orthopaedic surgeon and physical examination revealed point tenderness in the lumbar spine. Due to the new onset of low back pain, and concern for new compression fractures following the kyphoplasty, a new MRI was performed which documented acute compression fractures of the superior endplates of T10 and T11. Three weeks later, the patient underwent kyphoplasty of T10 and T11 and was started on IV bisphosphonate therapy with Reclast (zoledronic acid).

Two weeks following the kyphoplasty of T10 and T11, the patient felt a sudden pop in his back and experienced severe back pain once again. MRI documented acute compression fractures at T12 and L1. At this point, his brace was causing respiratory issues so it was discontinued and he underwent kyphoplasty of T12 and L1. Pain was still not relieved following this kyphoplasty, so one month later the patient underwent L3-S1 epidurals. This was without success, so a new MRI was completed which documented acute compression fractures at L3, L4, and L5. A cruciform anterior spinal hyperextension (CASH) brace was recommended, as the patient still could not tolerate a constrictive brace due to his COPD. Two weeks later, the patient underwent kyphoplasty of L4 and L5 followed by kyphoplasty of L2 and L3 another two weeks later.

The patient subsequently experienced pain relief for approximately seven months. However, after these seven months, upper back pain returned and radiographs documented a new compression fracture at T8 with 40% loss of height. Two weeks later, the patient underwent repeat kyphoplasty at T8. Several days later, he developed midback pain, and physical examination revealed tenderness in the midthoracic spine. X-rays documented a new compression fracture at T7, and the patient was instructed to continue to wear his brace. At follow-up, five weeks later, his pain was significantly improved, and the patient was tolerating the brace. X-rays documented no change of the compression fracture at T7, and the patient was instructed to continue wearing the brace.

**Figure 1:**
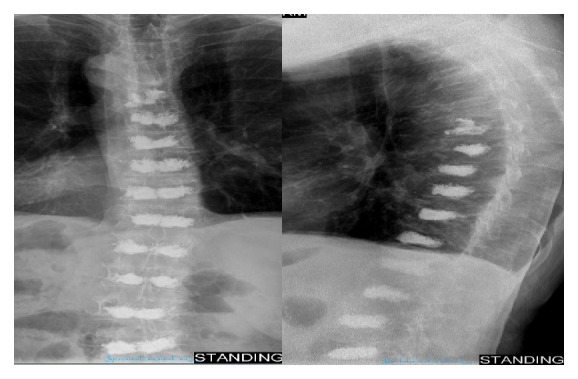
AP and lateral X-rays taken of the thoracic spine following the last of the eleven kyphoplasties. The patient underwent two kyphoplasties of T8 and one each of T9–T12.

**Figure 2:**
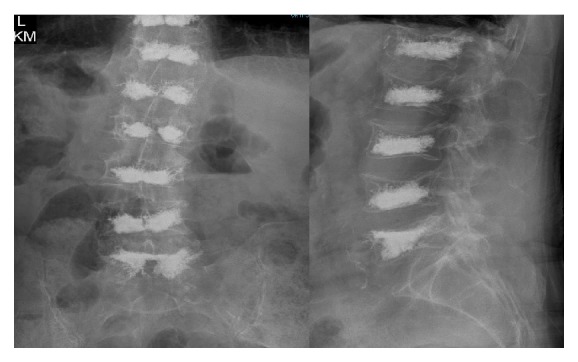
AP and lateral X-rays of the lumbar spine following the last kyphoplasty. The patient underwent one kyphoplasty of each lumbar vertebral body.

Figures 1 and 2 show the patient's thoracic and lumbar spines, respectively, following the last of the eleven kyphoplasties.

In summary, the patient underwent a total of eleven kyphoplasties, two in T8 and nine in T9-L5. The multiple kyphoplasties have allowed the patient to maintain his physiologic kyphosis. Before any vertebral compression fractures, his physiologic kyphosis was 40°. After completion of all eleven kyphoplasties, his kyphosis was maintained at 38°. This in turn prevented loss of lung volume and abdominal volume and worsening of his COPD. These procedures have allowed the patient to function without pain and maintain his employment.

## 3. Discussion

Vertebral kyphoplasty allows a patient to maintain vertebral height, restore the biomechanical balance of the spine, and prevent loss of lung and abdominal volume. The procedure can mitigate the negative effects of kyphosis on respiratory and digestive function, which have been well demonstrated in the literature. Lombardi Jr. et al. have found that negative effects on lung function occur when the angle of thoracic kyphosis reaches 55 degrees or greater, most notably decreased lung volume [8]. Correction of the deformity can improve lung volume as measured by forced vital capacity, though it may not increase the patient's forced expiratory volume measured in one second [9]. Gastroesophageal reflux has been linked to the presence of multiple vertebral fractures and kyphosis of the lumbar spine [10]. Lumbar kyphosis may specifically contribute to the symptoms by increasing intra-abdominal pressure, leading to acid reflux and hiatal hernia.

Kyphoplasty also prevents the continuation of microfractures through the vertebral body. However, complications include extrusion of cement into the canal and neurologic injury, infection, hematoma, PE, failure to relieve pain, and fracture of adjacent vertebral bodies. One study found that kyphoplasty can cause an increase of up to 19% in pressure of adjacent intervertebral discs. The increased strength of the index vertebral body can exaggerate force transmission to both adjacent and remote vertebral bodies leading to an adjacent level fracture [11].

Another study demonstrated that, after an osteoporotic related vertebral compression fracture, there is a 19% increase of an adjacent level fracture without surgical intervention [12]. After kyphoplasty is performed, the likelihood of an adjacent level fracture increases to 21% within sixty days, with the overall increase ranging from 3 to 29%. With vertebroplasty, the incidence ranged from 12 to 52% [12]. Furthermore, biomechanical studies demonstrated that kyphoplasty caused an increase in the vertebral body stiffness, which lead to a decrease in the ultimate load to failure of adjacent vertebrae by 8–30% [12]. With bipedicular filling, there was an 18% change in maximal principal strain of adjacent vertebrae in the inferior endplates in flexion; with unipedicular filling, the change was 11% [12].

Another study looked at adjacent level fractures in both osteoporotic patients and those on long term steroid therapy. In patients on long term steroid therapy, there was a 15% incidence of an adjacent level fracture per vertebral body undergoing kyphoplasty and a 22.6% incidence per patient [13]. For patients with primary osteoporosis, there was an 11.25% incidence of adjacent level fractures following kyphoplasty and a 24% incidence of fractures without kyphoplasty [13]. This study suggested that kyphoplasty did not necessarily increase the incidence of adjacent level fractures; rather, it led to a decrease in this population. This same study demonstrated that patients on long term steroid therapy had a 48% incidence of adjacent level fractures. The authors of this study concluded that long term steroid therapy leads to decreased bone formation, decreased intestinal absorption of calcium, and decreased proliferation of osteogenic precursors which significantly increases the risk of fractures [13]. The detrimental effects of long term steroid therapy are demonstrated in this patient who suffered multiple subsequent vertebral compression fractures following multiple kyphoplasties.

Furthermore, another study found a 10% incidence of adjacent level fractures within 90 days of a vertebral compression fracture and a 15% incidence overall. These authors concluded that age was not a significant factor in adjacent level fractures. Ten patients in this study were on long term steroid use and two of them developed fractures [14].

Although evidence favoring kyphoplasty has grown in the last few years, surgeons should exercise clinical judgment and evaluate each patient individually. Patient preference should also have a substantial influential role in the decision making process.
